# Understanding Echoics: Identifying Predictive Indicators of Vocal Imitation

**DOI:** 10.1007/s40616-024-00213-7

**Published:** 2024-12-10

**Authors:** Lee Mason, Alexis Bolds, Maeve Gavagan, Chris Ninness

**Affiliations:** 1 Child Study Center, Cook Children’s Health Care System, 1300 W Lancaster Ave, Fort Worth, TX 76110 USA; 2 Burnett School of Medicine, Texas Christian University, Fort Worth, TX USA; 3 College of Professional Education, Texas Woman’s University, Denton, TX USA; 4 Behavioral Software Systems & Human Interventions Institute, Nacogdoches, TX USA

**Keywords:** Binomial logistic regression, Language predictors, Verbal behavior, Autism spectrum disorder

## Abstract

A growing body of literature supports the use of echoic prompts toward conditioning other functional language skills. However, many individuals with autism spectrum disorder do not emit echoic behavior. Identifying the prerequisite skills of an echoic repertoire may be beneficial for intervention planning and clinical decision making. A chart review was conducted for 118 patients with autism who received early intensive behavioral intervention. We examined the Level 1 scores on the *Verbal Behavior Milestones Assessment and Placement Program* (Sundberg, 2014) for all patients. Using their scores on the echoic skills subtest as a dependent variable, we ran a binomial logistic regression in which the remaining eight domains, along with age and sex, served as independent variables. Our logistic regression model was statistically significant: *X*^*2*^(10) = 109.61, *p* < .001. The model explained 86.0% of the variance in echoic verbal behavior and correctly classified 95.8% of cases. Patients who demonstrated a mand repertoire were greater than 3.5 times more likely to also exhibit an echoic repertoire. Additionally, vocal play and motor imitation were associated with an increased ability to echo. Using binomial logistic regression, we identified three predictors influencing the echoic behavior of children with autism. Patients who demonstrated these three skills were statistically significantly more likely to echo the vocal verbal behavior of others. Additional research is now needed to confirm a functional relationship between each of these predictors and echoic control.

Echoic behavior is a formal subclass of intraverbal control (Vargas, 1982) in which point-to-point correspondence is shared with a precipitating verbal stimulus (Catania, 1998). Said again, the verbal response is duplicated (Blair & Farros, 2019; Michael, 1982). One of the earliest verbal operants to be acquired (Horne & Lowe, 1996), echoic control is foundational to more complex verbal behavior as the minimal echoic repertoire (i.e., phonemes) may be recombined to create novel and larger verbal responses (Daly et al., 2004). Moreover, echoic prompts are frequently used to condition other verbal operants, including mands, tacts, and intraverbals (Kodak & Clements, 2009; Roncati et al., 2019).

Echoic responding typically emerges within the child’s first year of life (Pelaez et al., 2011; Poulson et al., 1991), contingent upon the shaping of speech sounds and selection by their verbal community (Horne & Lowe, 1996). Accordingly, the ability to echo is commonly used as an early marker for identifying children at risk of developing autism spectrum disorder (ASD; Neimy et al., 2020). Among the heterogeneous population of individuals with ASD, 25% to 35% have limited vocal repertoires (Rose et al., 2016). Parents frequently report language delays as their primary concern for seeking an ASD diagnosis (Lord et al., 2004). The failure to develop functional vocal language has been attributed to a paucity of resources available to families combined with a lack of direct services, and a dearth of research on individuals with profound ASD (Thurm et al., 2022). Currently, there are no reliable predictors to indicate which children with autism are likely to develop a vocal verbal repertoire. Identifying the factors that lead to the development of echoic control over verbal behavior may allow for targeted interventions to remediate the language deficits associated with ASD.

Using various statistical techniques, researchers have previously sought to identify variables that serve as predictors of later language development. A confounding factor in this pursuit is the lack of consensus regarding the terminology for “nonverbal” and “minimally verbal” (Koegel et al., 2020), which fails to distinguish between verbal behavior and vocal speaking; the latter being a subset of the former. Understandably, these studies have found a variety of putative predictors of vocal language as diverse as age (Fenske et al., 1985), sex (Carter et al., 2007), and pretend-play skills (Smith et al., 2007). A child’s baseline language inventory is most frequently reported as a significant factor, indicating that children who emit a broad range of vocalizations are more likely to develop functional vocal speech (Broome et al., 2022; Smith et al., 2007).

However, conflicting outcomes have shown that children with ASD may be “hypervocal” in comparison to their neurotypical peers (Swanson et al., 2018) and that elevated levels of vocal stereotypy may actually impede the development of verbal behavior (Wang et al., 2020). Notably, Warren and colleagues (2009) found that neurotypical children emitted significantly more verbal behavior (i.e., vocalizations consequated by a listener) than vocal stereotypy, while children with ASD emitted higher levels of vocal stereotypy than verbal behavior. This finding suggests that researchers might examine the contingencies of reinforcement responsible for language development rather than focusing on structural variables (e.g., age, sex) or specific response topographies (i.e., consonant and phonemic inventories).

Cividini-Motta and colleagues (2017) described three primary methods of explicitly reinforcing the verbal behavior of children with ASD. Stimulus-stimulus pairing is frequently used to condition vocalizations as a reinforcer (Esch et al., 2005). Sounds made by the child are paired with reinforcers before being brought under the discriminative control of an imitative verbal stimulus. Alternatively, vocal imitation training is used to shape vocalizations through the differential reinforcement of successive approximations to an imitative verbal stimulus (Carroll & Klatt, 2008). During vocal imitation training, the therapist models a specific vocal response and delivers reinforcement contingent on the child’s imitation of said response.

Finally, mand-model procedures are used during natural environment training by converging mand and echoic control (LeBlanc et al., 2006). “The infant's initial reflexive cries are rapidly converted to mands when reinforced by the responses of the parent,” explain Drash and colleagues (1999, p. 31). “After children begin to produce a variety of different sounds as verbal mands, the parent assigns words to these sounds” (p. 32). By reinforcing mands, the child may begin attending to other features of the environment to acquire new mands. Accordingly, mand training may establish transitive conditioned motivating operations for echoics (Ban & McGill, 2023). Despite subtle distinctions across these three practices, no single teaching procedure has been found to be reliably effective for conditioning echoic control over the verbal behavior of children with ASD (Cividini-Motta et al., 2017).

Given the importance of echoic control toward conditioning more complex verbal operants, it would be helpful to better understand the prerequisite learning histories necessary to support the occurrence of imitative vocal speech. Ackley and colleagues (2019) identified 18 different assessments and curriculum guides based on the principles of applied behavior analysis that could be used to identify potential predictive variables. Of these, the *Verbal Behavior Milestones Assessment and Placement Program* (VB-MAPP; Sundberg, 2014) is the most widely implemented language assessment, used by more than 75% of EIBI practitioners (Padilla, 2020). The basic skill domains within the VB-MAPP may provide a foundation for identifying the variables that contribute significantly toward the development of echoics.

Predictive analytics involves the use of statistical modeling to predict the value of an outcome variable on the basis of a series of predictor variables. When the outcome variable being predicted is dichotomous (i.e., the presence or absence of echoics), binomial logistic regression is frequently used owing to its strong explanatory power. Specifically, the significance of each independent variable is evaluated and assigned a probability value (*p* value). Moreover, logistic regression converts the binary output to a logit function for more intuitive interpretation.

The purpose of the current project was to use the VB-MAPP as a basis for predicting whether children with ASD would display an echoic repertoire. After compiling the VB-MAPP scores from multiple children with ASD, a logistic regression model allowed us to identify a *p* value and logit for each of the domains on the assessment. Consequently, we were able to identify relevant variables that contribute to the development of echoic control.

## Method

### Participants and Setting

We conducted a chart review of 118 young children with ASD who received early intensive behavioral intervention across a seven-year period between 2015 and 2022. As part of the comprehensive intervention provided by a not-for-profit children’s healthcare center, a VB-MAPP (Sundberg, 2014) assessment was administered to each patient every six months. Patients were 80% male, 20% female, and ranged from 1 year, 10 months to 8 years, 11 months of age at the time of initial assessment. Prior to intake, each patient had been independently diagnosed with ASD. No patients for whom an intake VB-MAPP was completed were excluded from this analysis.

All VB-MAPP assessments were completed in a clinical setting by a team of Registered Behavior Technicians under the supervision of a Board Certified Behavior Analyst. Following the scoring guidelines for the assessment, patients were either directly observed or tested for their ability to complete each of the skills included on the VB-MAPP. The initial assessment commenced immediately upon each patient’s admission to the outpatient center and was completed within a few days depending on the complexity of the patient’s skills. On their initial intake assessment, patient scores ranged from Level 1 to Level 3 across the various domains of the VB-MAPP. Patient records were maintained in an online database that – subsequent to receiving IRB approval – was accessed to create a de-identified dataset for our analyses.

Notably, many of the participants whose records were accessed for this research communicated using speech-generating devices (SGDs). These patients were not necessarily non-vocal, as SGDs are commonly used to help support vocal speech (Carnett & Ingvarsson, 2016). Moreover, the vast majority of patients who used SGDs did not begin doing so until after their initial intake assessment. The decision to assign them an SGD was based in part on these scores.

### Measure

The VB-MAPP is a criterion-referenced assessment of basic language and language-related skills that is commonly used to assess the communication and social-skills deficits of children with ASD. The VB-MAPP Milestones Assessment contains 170 language and collateral milestones that are sequenced across three developmental levels. Given the purpose of identifying predictors of echoic behavior, we were exclusively interested in the data from Level 1 of the initial VB-MAPP administration that was collected at the onset of services for each child. Level 1 consists of nine domains balanced across a scale of 1 to 5, which correspond with the language skills acquired by typically developing children from birth to 18 months of age.

Embedded within the VB-MAPP is a sub-test of verbal imitation called the *Early Echoic Skills Assessment* (EESA; Esch, 2008) that is used to rate the speaker’s ability to echo a variety of speech phonemes, syllable combinations, and intonation patterns. The EESA comprises five groups of test items worth a total of 100 points. The score for the echoic domain of the VB-MAPP is determined by how many points are scored on the EESA. For example, two points on the EESA equates to one point on the echoic domain, five points on the EESA equates to two points on the echoic domain, 10 points on the EESA equates to three points on the echoic domain, 15 points on the EESA equates to four points on the EESA, and 25 points on the EESA – of which 20 must come from group 1 – equates to five points on the echoic domain. The remaining 75 points on the EESA correspond to Level 2 of the VB-MAPP's echoic domain.

### Dataset

Compiling the data from the initial administrations of the VB-MAPP Milestones Assessment for each patient, we developed a dataset of 118 rows that consisted of the patient’s age (at the time of initial assessment), sex, and the score recorded for each of the nine domains from Level 1: Mand, Tact, Listener Responding, Visual Perceptual Skills and Matching to Sample (VP-MTS), Independent Play, Social Behavior and Social Play, Motor Imitation, Spontaneous Vocal Behavior, and Echoic, as demonstrated by the EESA. The score for each domain ranged from 0 to 5 in half-point increments. According to Sundberg (2014), the scores across domains are standardized, such that a Mand score of 5 is developmentally equivalent to a score of 5 in each of the remaining eight domains of Level 1. A neurotypical child, therefore, would show similar scores across each domain. However, prior research on individuals with ASD has shown circumscribed learning patterns that may result in isolated behavioral excesses or deficits (Ploog, 2010). In contrast to their typically developing peers, a high score in one domain of the VB-MAPP is not necessarily representative of high scores in the other domains.

### Variables

Along with each patient’s age and sex, we examined their scores on Level 1 of the VB-MAPP Milestones Assessment. The nine domains assessed within Level 1 are designed to correspond with the skills displayed by neurotypical children up to 18 months of age. Five of these domains are designed to assess functional (i.e., mand, tact, listener, and echoic) and topographical (i.e., vocal) properties of language.

Level 1 of the Mand domain ranges from emitting two mands with the assistance of imitative or echoic prompts (1 point) to emitting 10 mands in the presence of the specified reinforcer (5 points).The Tact domain ranges from emitting two tacts with the assistance of imitative or echoic prompts (1 point) to emitting 10 different tacts (5 points). The Listener domain ranges from consistently making eye contact with a speaker (1 point) to selecting 20 named items from an array (5 points). The Vocal domain ranges from spontaneously emitting five sounds within an hour (1 point) to spontaneously emitting 15 different identifiable words within an hour (5 points). As noted above, the Echoic domain ranges from scoring at least two on the EESA subtest (1 point) to scoring at least 25 on the EESA subtest (5 points).

Notably, the language skills assessed within Level 1 of the VB-MAPP are not mutually exclusive. For example, the first Mand and Tact milestones allow the use of echoic prompts, while the second Mand milestone allows the presence of the desired item (i.e., Tact). The interaction of these basic language skills has been well documented within the verbal behavior literature (Mason et al., 2024; Michael et al., 2011).

The remaining four domains are described by Sundberg (2014) as language-related skills. The VP-MTS domain ranges from visually tracks moving stimuli for 2 s (1 point) to matching 10 identical items (5 points). The Play domain ranges from interacting with objects for one minute (1 point) to independently engaging in cause-and-effect play for two minutes (5 points). The Social domain ranges from making eye contact as a type of mand (1 point) to spontaneously imitating the motor behavior of peers twice (5 points). Finally, the Imitation domain ranges from imitating two gross motor movements (1 point) to imitating 20 motor movements (5 points).

The EESA subtest was used as the binary dependent variable for this investigation. Verbal imitation skills typically emerge by 11 months of age and are frequently considered a prerequisite to more complex verbal behavior (Esch, 2008). The EESA is designed to evaluate a person’s ability to echo the language models provided by another person and is completed in full with each administration of the VB-MAPP.

For the current experiment, the presence or absence of an echoic repertoire served as the dependent variable, while the remaining eight domains of Level 1 of the VB-MAPP served as independent variables. We designed the study to assess the extent to which the scores of these eight surrounding domains are predictive of an echoic repertoire using a logistic regression model that employed a binary dependent variable. Prior to conducting the analyses, we determined a threshold of five points on the EESA as the cut point for distinguishing between participants with and without an echoic repertoire. That is, participants who scored less than five on the EESA subtest (i.e., < 2 points on the Echoic domain) were classified as demonstrating no echoic repertoire (0). Participants who scored five or above on the EESA subtest (i.e., ≥ 2 points on the Echoic domain) were classified as demonstrating an echoic repertoire (1).

**Fig. 1:**
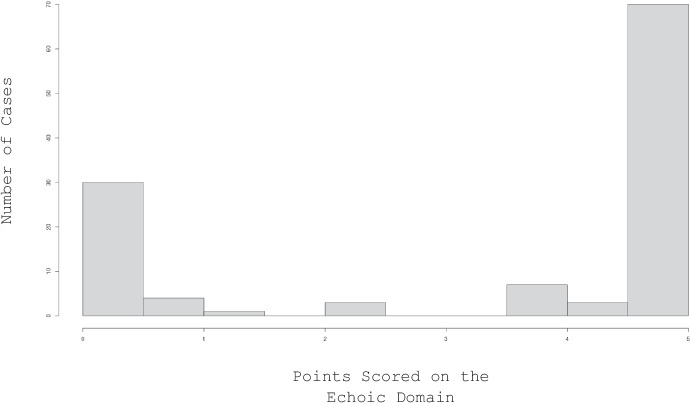
The distribution of patient scores in the echoic domain

Figure 1 shows the distribution of Echoic domain scores for the 118 patient participants. Two natural breakpoints are discernable in which no patients received scores, 1.5–2.0 and 2.5–3.5. We used these breaks to determine the assignment of patients to the two outcome groups.

As noted above, the VB-MAPP assessments were completed immediately upon each patient’s admission to the clinic. The presence of a novel person often has the effect of artificially suppressing the behavioral repertoires of young children; an effect of inadequate rapport building (Gavett & McCaffrey, 2007). Consequently, it is possible that many children have stronger echoic repertoires than are indicated by their initial VB-MAPP assessments. We hypothesized that any abative effect of the assessment conditions was likely to completely suppress the child’s echoic repertoire. A four-point maximum on the EESA allowed for partial, but certainly not generalized, echoic control. Rather than only including “true zeros” within this group, we wanted to reduce the likelihood of a type II error by concluding the patient does not reliably echo the verbal behavior of others when – in their natural environment – they may.

Conversely, it is unlikely that a false positive, in which a child is scored as having a stronger echoic repertoire than they demonstrate in everyday circumstances, would be identified. We selected the lower, more conservative of the two breakpoints in Fig. 1 as the threshold for an echoic repertoire to better isolate the emergence of echoic control. To the extent that other domains on the VB-MAPP help strengthen an already existing echoic repertoire, our focus on the emergence of echoics would be obfuscated.

### Interobserver Agreement

Interobserver agreement (IOA) was assessed by asking a second rater to independently re-code 50% (*n* = 59) of the records used in our analysis. The IOA sample was randomly selected by assigning each record a random number. Next, the dataset was sorted by random number in ascending order. We then renumbered the dataset sequentially from 1 to 118 and extracted the odd-numbered records for IOA. Of the 59 records that were re-coded, 55 (93%) showed 100% agreement on the scores recorded for each VB-MAPP domain. For the remaining four records, point-by-point agreement across each of the domains averaged 86%, range 78% (agreement on 7/9 domains) to 89% (agreement on 8/9 domains). These four records were then reviewed a third time to resolve the disagreement.

### Data Analysis

Using SPSS 25, we performed a binomial logistic regression to ascertain the effects of age, gender, and VB-MAPP Level 1 scores on the likelihood that participants will demonstrate an echoic repertoire as measured by the Early Echoic Skills Assessment (EESA; Esch, 2008). All continuous independent variables were found to be linearly related to the log-odds transformation (logit) of the dependent variable (Box & Tidwell, 1962). Of the 118 participants, we identified two standardized residuals with values greater than two standard deviations, − 6.46 and 2.97. Given the heterogeneity of ASD, we elected to include both of these cases in our analysis.

## Results

Table 1 provides descriptive statistics and correlation for the nine domains that comprise Level 1 of the VB-MAPP. Stronger correlations (> 0.80) were found among all three verbal operants: Mand, Tact, and Echoic. Moderate correlations (0.50–0.80) were found between VP-MTS, Listener, Imitation, and Vocal domains, as well as between these domains and the verbal operants. Weaker correlations (< 0.50) were found for Play and Social domains (with the exception of Social ~ VP-MTS, for which a moderate correlation was identified).

**Table 1.**
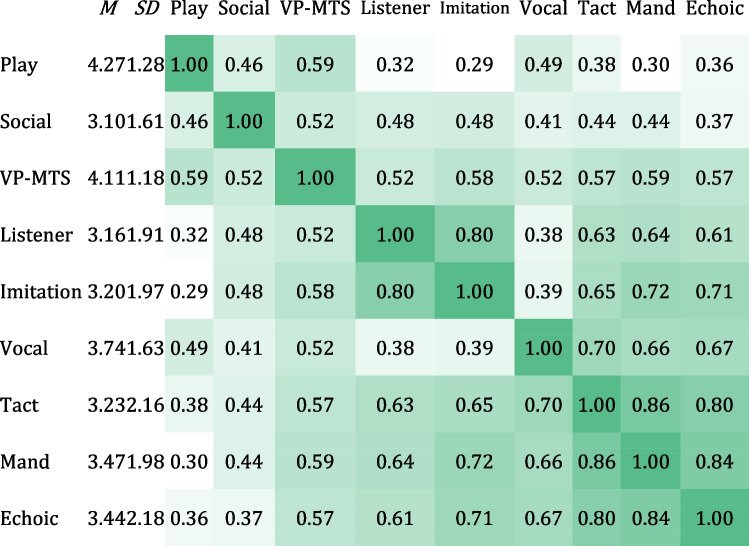
A correlation matrix showing the relationship between VB-MAPP domains

**Fig. 2:**
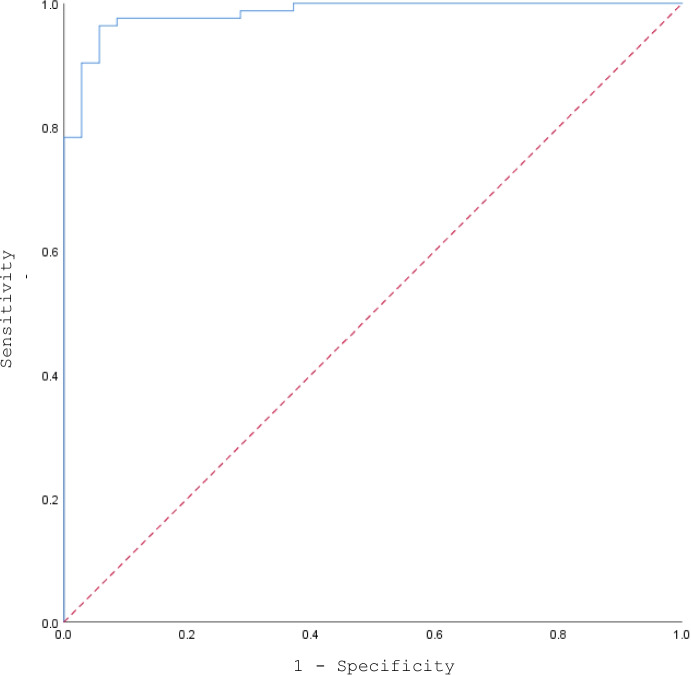
A ROC curve of the logistic regression model’s predictive findings

The logistic regression model was statistically significant, *X*^*2*^(10) = 109.61, *p* < 0.001. The model explained 86.0% of the variance in echoic control (Nagelkerke *R*^*2*^) and correctly classified 95.8% of cases.^1^ Sensitivity, the proportion of cases accurately classified as demonstrating an echoic repertoire, was 96.4%. Specificity, the proportion of cases accurately classified as demonstrating no echoic repertoire, was 94.3%. Positive predictive value, the proportion of cases predicted to demonstrate an echoic repertoire, was 97.6%. Negative predictive value, the proportion of cases predicted to demonstrate no echoic repertoire, was 91.7%. Figure 2 shows that the area under the receiver operating characteristic (ROC) curve was 0.984, 95% CI [0.967, 1.00], which is considered an outstanding level of discrimination for the predictive model (Hosmer et al., 2013).

Three of the predictor variables from Level 1 of the VB-MAPP were found to be statistically significant additions to the model: Mand, Vocal, and Imitation (see Table 2 below). For each one-unit increase in the value of the Mand repertoire, participants were 3.54 times more likely to exhibit echoic control. Similarly, increased scores in the Vocal domain were associated with a 3.36-fold increase in echoic control, while increased scores in the Imitation domain were 2.50 times more likely to display an echoic verbal repertoire.

**Table 2:** Logistic regression predictions of an echoic verbal repertoire

	*B*	SE	Wald	*df*	*p*	Odds ratio	95% CI for odds ratio	
							Lower	Upper
Age	.12	.31	.15	1	.706	1.12	.62	2.05
Gender	2.40	1.32	3.29	1	.070	11.04	.82	148.02
Mand	1.27	.48	7.00	1	.008**	3.54	1.39	9.05
Tact	− .51	.41	1.55	1	.213	.60	.27	1.34
Listener	− .02	.40	.00	1	.969	.98	.45	2.17
VP/MTS	.26	.45	.32	1	.569	1.29	.53	3.14
Play	.19	.43	.19	1	.665	1.21	.52	2.80
Social	− .57	.36	2.47	1	.115	.57	.28	1.15
Imitation	.92	.45	4.09	1	.043*	2.50	1.03	6.07
Vocal	1.21	.49	6.01	1	.014*	3.36	1.28	8.84
Intercept	− 7.28	2.81	6.72	1	.010	.00		

^*^p < .05. **p < .01

Level 1 scores on the remaining domains of VB-MAPP (i.e., Tact, Listener, VP/MTS, Play, and Social) were not associated with echoic verbal behavior. Age also did not contribute to the predictive model. Gender, boys compared to girls, approached significance but fell outside of the critical region.

## Discussion

The results of a binomial logistic regression identified three independent variables that increase the likelihood of an individual with ASD emitting echoic behavior: scores on the Vocal, Imitation, and Mand domains of the VB-MAPP. Our results confirm and expand upon the putative predictors of speech identified in prior research. Unlike prior research in this area that based speech predictions on norm-referenced assessments of expressive language, our analysis incorporated a criterion-referenced test that measured performance across functionally distinct verbal operants.

The findings of our logistic regression are consistent with prior research to establish echoic control over the verbal behavior of speakers with ASD. Specifically, each of the three statistically relevant VB-MAPP domains – Vocal, Imitation, and Mand – correspond with methods of reinforcing echoic behavior found in the behavior-analytic literature: stimulus-stimulus pairing, vocal imitation training, and mand-model procedures, respectively.

The significance of the Vocal domain identified by our logistic regression model supports prior research on stimulus-stimulus pairing to increase vocalizations by conditioning them as a reinforcer (Carroll & Klatt, 2008; Miguel et al., 2002). Researchers have previously identified an individual’s linguistic inventory as an important predictor of vocal speech (Broome et al., 2022; Smith et al., 2007). Although the VB-MAPP does not include a comprehensive phonemic inventory, the Vocal domain is designed to measure a participant’s spontaneous vocal behavior. Specifically, the milestones within this domain sample the frequency and variability of sounds and words emitted by the participant within a timed observation of 60 min. Vocalizations such as babbling are important to developing speech, because, in addition to strengthening the vocal musculature, vocal play produces sounds that are likely to be repeated, varied, and ultimately selected (Abreu-Rodrigues et al., 2005). Moreover, to the extent that the speaker’s vocalizations achieve parity with those of the verbal community, spontaneous vocal play may be automatically reinforced (Palmer, 1996).

The Imitation domain was also found by our model to be an important predictor of Echoics. More than half a century ago, Baer and colleagues (1967) demonstrated the use of generalized motor imitation to establish vocal imitation. “In these applications, the stimulus class of behavioral similarity was, in numerous examples, made discriminative with respect to positive reinforcement. Hence, similarity could be expected to take on a positive reinforcing function as well as a discriminative function” (Baer et al., p. 415). Subsequent research has shown that a generalized motor imitation repertoire can be transferred to vocal imitation. For example, Ross and Greer (2003) presented a high-probability motor imitation sequence prior to an imitative vocal model to induce echoic behavior. Imitation is assessed across Levels 1 and 2 of the VB-MAPP, which samples gross and fine motor imitation, imitation with objects, spontaneity, conditional discriminations, sequences of actions, functional skills, and novel actions. A generalized imitative repertoire is important to speech because it helps to condition other people’s behavior, both motor and verbal, as discriminative stimuli.

Finally, our model showed that the Mand domain, a functionally distinct verbal operant, contributed significantly to Echoic control. As noted above, researchers have indicated that echoic behavior is a helpful form of supplemental stimulation for mand training, a critical component of EIBI (Kodak & Bergmann, 2020). However, research on mand-model procedures has demonstrated the effects of utilizing an existing mand repertoire to help establish echoic behavior (Drash et al., 1999). An individual’s ability to mand is assessed across all three levels of the VB-MAPP, which samples the variety, generality, novelty, and complexity of mands to access tangibles, information, escape, and attention. The importance of a functional mand repertoire is important to echoic behavior for two primary reasons, explain Drash et al. (1999):

First, by reinforcing an acceptable vocal mand repertoire, the child’s vocal repertoire is altered in that almost any reinforcer can be obtained during therapy by simply emitting a brief nonaversive vocal response. The tendency to emit acceptable verbal behavior is thus strengthened well before imitation training begins. Second, by initially using the child’s sounds as prompts during mand training, the sounds become multiply controlled as both mands and echoics, thus increasing the probability that the child will imitate during echoic training (p. 41).

While echoic behavior is commonly used to help establish a mand repertoire, our results support those of Drash and colleagues to indicate that mands are critical to developing imitative verbal behavior.

The results of this study point to different areas in which additional research is necessary to better understand the language development of individuals with ASD. Specifically, we have identified three independent variables predictive of echoic behavior, but no functional relationships. These three variables – mand, motor imitation, and vocal skills – each have their own prerequisite foundations that may lead to additional predictive variables. In addition to replicating the results of the current study, researchers should look at systematically manipulating each of these variables, independently and in combination, toward the establishment of echoic behavior for individuals with ASD.

A primary benefit of logistic regression is the inclusion of odds-based outcomes when interpreting results. Specifically, the *Odds Ratio* column in Table 2 indicates the change in the dependent variable (i.e., score on Echoic domain) for each increase in one unit of the corresponding independent variable. Although our echoic data were coded as a binary variable for building our statistical model, the logit transformation converts them back to a continuous variable for interpretation.

First, our results showed that for every one-point increase in the Vocal domain, Echoic scores increased by 3.36. Future research should more directly examine the relationship between an individual’s phonemic inventory and their echoic repertoire. For example, researchers can examine the extent to which reinforcing a greater variety of speech sounds impacts echoic control (see Gevarter & Horan, 2018).

Second, our results indicated that for every one-point increase in the Imitation domain, Echoic scores increased by 2.50. Future research should seek to better understand the relationship between motor and vocal imitation. While Baer and colleagues (1967) established a precedent for this line of research, little has been done in the past 50 years to replicate and expand upon these findings. Espanola Aguirre and Gutierrez (2019) proposed a hierarchy of bodily, vocal, and facial imitation skills, but additional research on auditory-visual conditional discrimination is necessary to bridge the gap between motor and vocal imitation (see Carp et al., 2015). Recent findings by Pittet et al. (2022) highlighted the potential for motor imitation to serve as a predictor of expressive language development with a greater effect on vocal repertoire in children with more robust motor imitation skills at baseline. In light of our findings and the growing significance of motor skills and imitation on vocal repertoire (i.e., speech production), future research should aim to better understand the relationship between that of gross and fine motor skills and imitation on echoic repertoire.

Finally, our results found that for every one-point increase in the Mand domain, Echoic scores increased by 3.54. Additional research should aim to establish a functional relationship between an individual’s mand and echoic repertoire. Though Drash et al. (1999) first proposed using an individual’s mand repertoire to condition echoic control, the preponderance of research has focused on the reverse. As noted by Tincani and colleagues (2020), the majority of current literature on SGD use in children with ASD primarily targets mand repertoire, with rare exceptions focusing on other verbal operants. Researchers should, therefore, continue to examine how SGDs and other forms of augmentative and alternative communication may be used to establish an echoic repertoire (see Cividini-Motta et al., 2017).

A primary limitation to this research is the multicollinearity of independent variables in our analysis. The interdependence of verbal operants is well established within the literature (Mason et al., 2024; Michael et al., 2011), and it should therefore be expected that correlations exist between and across the language and language-related skills on the VB-MAPP. However, multicollinearity challenges the interpretation of our results. For instance, the above-noted 1:3.54-point Mand-to-Echoic ratio assumes that all other independent variables are held constant. Our understanding of mand control as a behavioral cusp triggering second- and third-order contingencies allows us to anticipate changes across other domains (Becker et al., 2024). Pragmatically speaking, a concomitant increase across multiple domains should be welcomed, but it is up to future researchers to pinpoint functional relationships between independent and dependent variables.

While the current research focused on identifying relevant predictors of echoic control, future research should continue to examine additional predictors and prerequisites of verbal behavior. Given the significance of the Mand domain identified here, additional research to identify the predictors of mand control seems warranted. We also noted that a multitude of statistical approaches have previously been used to identify predictors of language. Binomial logistic regression is just one method of modeling the verbal repertoire. Other researchers may seek to validate our results using additional statistical methods.

Certain methodological factors impacted the results of this study. First, we constrained our independent variables to Level 1 of the VB-MAPP. Given our focus on identifying predictive variables of echoic control, along with the developmental layout of the VB-MAPP, we did not consider more advanced skills as potential predictors. Considering the idiosyncratic developmental patterns of ASD, however, future researchers should consider examining data from Levels 2 and 3, in addition to other features of the VB-MAPP (e.g., Barriers Assessment).

Additionally, our criterion for determining whether or not patients demonstrated echoic control undoubtedly affected our results. We selected 5 points on the EESA as the threshold, as it allowed for a modicum of, though far from generalized, echoic control. More stringent or relaxed interpretations of this variable may lead to different outcomes and should be evaluated through systematic replication.

Multiple literature reviews have noted research on echoic behavior is accumulating at a slower rate than research on other elementary sources of vocal verbal behavior (DeSouza et al., 2017; Petursdottir & Devine, 2017). We extended the research on predictive models of vocal language by employing binomial logistic regression to identify three predictors of echoic behavior. Our hope is that these findings re-energize additional research on this fundamental verbal operant to help the scientific community better understand why children with ASD fail to develop vocal verbal behavior.

## Data Availability

The data that support the findings of this study are available from the corresponding author upon reasonable request.

## Footnotes

1 When the two outliers were removed, the model correctly classified 100% of cases.
